# CRX Is a Diagnostic Marker of Retinal and Pineal Lineage Tumors

**DOI:** 10.1371/journal.pone.0007932

**Published:** 2009-11-20

**Authors:** Sandro Santagata, Cecile L. Maire, Ahmed Idbaih, Lars Geffers, Mick Correll, Kristina Holton, John Quackenbush, Keith L. Ligon

**Affiliations:** 1 Department of Pathology, Brigham and Women's Hospital, Boston, Massachusetts, United States of America; 2 Department of Pathology, Children's Hospital Boston, Boston, Massachusetts, United States of America; 3 Department of Pathology, Harvard Medical School, Boston, Massachusetts, United States of America; 4 Department of Medical Oncology and Center for Molecular Oncologic Pathology, Dana-Farber Cancer Institute, Boston, Massachusetts, United States of America; 5 Department of Genes and Behavior, Max-Planck-Institute of Biophysical Chemistry, Goettingen, Germany; 6 Center for Cancer Computational Biology, Dana-Farber Cancer Institute, Boston, Massachusetts, United States of America; Katholieke Universiteit Leuven, Belgium

## Abstract

**Background:**

CRX is a homeobox transcription factor whose expression and function is critical to maintain retinal and pineal lineage cells and their progenitors. To determine the biologic and diagnostic potential of CRX in human tumors of the retina and pineal, we examined its expression in multiple settings.

**Methodology/Principal Findings:**

Using situ hybridization and immunohistochemistry we show that Crx RNA and protein expression are exquisitely lineage restricted to retinal and pineal cells during normal mouse and human development. Gene expression profiling analysis of a wide range of human cancers and cancer cell lines also supports that *CRX* RNA is highly lineage restricted in cancer. Immunohistochemical analysis of 22 retinoblastomas and 13 pineal parenchymal tumors demonstrated strong expression of CRX in over 95% of these tumors. Importantly, CRX was not detected in the majority of tumors considered in the differential diagnosis of pineal region tumors (n = 78). The notable exception was medulloblastoma, 40% of which exhibited CRX expression in a heterogeneous pattern readily distinguished from that seen in retino-pineal tumors.

**Conclusions/Significance:**

These findings describe new potential roles for CRX in human cancers and highlight the general utility of lineage restricted transcription factors in cancer biology. They also identify CRX as a sensitive and specific clinical marker and a potential lineage dependent therapeutic target in retinoblastoma and pineoblastoma.

## Introduction

Pineal parenchymal tumors predominantly affect children, and account for approximately one-quarter of all neoplasms of the pineal region [1]. These tumors exhibit a spectrum of clinical aggressiveness that include pineocytomas, which are low-grade well-differentiated and indolent tumors often with large pineocytomatous rosettes; pineoblastomas, which are high-grade poorly-differentiated aggressive embryonal tumors with dense sheets of poorly differentiated small cells and pineal parenchymal tumors of intermediate differentiation (PPTID), which have an intermediate grade and prognosis[2]–[7]. The appropriate pathologic classification and grading of tumors of the pineal region is essential for determining clinical management and prognosis[8], however, the diagnostic evaluation is often difficult due to the inherently small size of the biopsies for diagnosis and the wide array of tumor types that can involve the pineal gland[3], [9]. The most common tumors entering the differential diagnosis are CNS germ cell tumors, primitive neuroectodermal tumors, gliomas, atypical teratoid/rhabdoid tumors and anaplastic ependymoma[2], [6], [10]. However, specific markers which can positively identify all pineal lineage tumors are generally lacking in clinical practice. In addition, research into the biology and treatment of these neoplasms has been severely hindered by the rare nature of the tumors, the lack of primary tissue available for study, and the scarcity of relevant cell lines or mouse models of the disease. Each of these research areas would greatly benefit from the discovery of reliable markers of the disease.

The pineocytes of the pineal and the cone and rod photoreceptors of the retina share histological, ultrastructural, immunohistochemical and pathologic features. Histologically, the human pineal gland shows rosettes resembling those of the developing retina[11]. Ultrastructurally evaluation of pineal parenchymal tumors variably reveals some evidence of photoreceptor differentiation including bulb-ended cilia with a 9+0 axial skeleton protruding into an intracytoplasmic lumen, microtubular sheaves, and vesicle-crowned and annulate lamellae [12]–[15] but such features are not present reliably enough for routine clinical diagnosis. Pineal parenchymal tumors have been shown to express antigens found in the retina including retinal S-antigen[16], [17], transducin[18], [19], and interphotoreceptor retinoid-binding protein, rod opsin, cone opsin, and cellular retinaldehyde-binding protein[20]. Conversely, normal human retina and retinoblastoma express retinal and pineal antigens consistent with incomplete retinal lineage differentiation, and a bias towards cone photoreceptor antigens[21]. The common lineage connection between the pineal and retina is further exemplified by the occurrence of pineoblastoma in patients with retinoblastoma, a phenomenon termed trilateral retinoblastoma[22]–[24]. This shared heritage strongly suggests that lineage-restricted biomarkers found in the developing retina and pineal may be useful not only as immunohistochemical markers in the diagnosis of retino-pineal tumors but possibly in the etiology or treatment of these tumors.

As a class, transcription factors are emerging as highly reliable tools in the pathologic diagnosis of human solid tumors[25]. Recently, our group and others demonstrated that lineage-restricted transcription factors such as OCT4 and NANOG are robust markers for the diagnosis of germ cell tumors, including those in the central nervous system[26]–[29]. Crx is an Otx-like homeobox transcription factor critical for photoreceptor differentiation and for maintenance of the transcriptional regulatory networks essential for normal retinal development [30]and pineal function[31]–[33]. Mutations in the human *CRX* gene lead to photoreceptor degeneration and the retinal diseases cone-rod dystrophy 2 (CORD2), Leber congenital amaurosis type VII (LCA7), and retinitis pigmentosa, late onset dominant[34]–[38]. Consistent with these findings, Crx null mice demonstrate a lineage dependent role for this TF in proper development of retinal stem/progenitor cells leading to susequent photoreceptor degeneration[39]. In addition, while the pineal gland appears grossly normal in post-natal Crx null mice, pineal-specific gene expression is reduced and circadian entrainment is attenuated[39]. Little is known about CRX expression or function in human cancer, although several studies have described its expression in retinoblastoma cell lines. Given its restricted expression and functional relevance in pineal and retinal cell lineages we sought to more comprehensively establish whether CRX might serve as a robust TF marker for research and digansotic evaluation of retino-pineal tumors.

In this study we demonstrate expression of Crx in normal and neoplastic cells of retinal and pineal lineage and demonstrate the utility of immunohistochemistry for Crx in discriminating pineal parenchymal tumors from other lesions that often enter the differential diagnosis of pineal masses.

## Materials and Methods

### Ethics Statement

This study was conducted according to the principles expressed in the Declaration of Helsinki. All work on human tissues was conducted on anonymous excess archival human material from the Departments of Pathology at Children's Hospital Boston and Brigham and Women's Hospital. The research study was approved by the Children's Hospital Boston Institutional Review Board for Human Research and also the Brigham and Women's Hospital Institutional Review Board for Human Research as an excess tissue protocol. The data were analyzed anonymously and therefore both review boards did not require specific written consent from patients for this study.

### Tissue Samples

Paraffin blocks from surgical resection specimens spanning a 10 year period (1998–2008) were obtained as anonymous specimens from Children's Hospital, Boston and Brigham and Women's Hospital, Boston, in accordance with the regulations of the review boards of both institutions for excess tissue. Diagnoses were confirmed based on World Health Organization diagnostic criteria. Surgical resection samples consisted of five normal pineal tissue, three pineal cysts, five pineoblastoma, four pineocytoma, four pineal parenchymal tumor of intermediate differentiation, nine CNS germinoma, four CNS embryonal carcinoma, ten medulloblastoma, five supratentorial primitive neuroectodermal tumor, five atypical teratoid/rhabdoid tumor, five Langerhan's cell histiocytosis, five neurocytoma, 12 glioblastoma, 12 anaplastic oligodendroglioma, five meningioma, five choroid plexus carcinoma, six anaplastic ependymoma, five metastatic carcinoma (one lung adenocarcinoma, one ductal carcinoma of the breast, one neuroendocrine carcinoma, one renal cell carcinoma and one melanoma) and enucleation specimens of retinoblastoma. Paraffin blocks of ten pineals from post-mortem examination were also obtained from the archives of Brigham and Women's Hospital. The pineal tumor samples were consecutive samples that had sufficient tissue present in the block for research use. The study was designed in light of recommendations from the STARD (STAndards for the Reporting of Diagnostic accuracy studies) statement. http://www.stard-statement.org/

### Slide Preparation, Immunohistochemistry and Scoring

Specimens were fixed in 10% buffered-formalin, four-micron sections were generated from paraffin blocks and slides were stained with hematoxylin and eosin (H&E). Serial sections of the paraffin blocks were cut and these slides were used for immunohistochemical studies. The antigen, clone, dilution, antigen retrieval conditions and vendors of the primary antibodies are listed in Table 1 and all antibodies are publicly available through commercial souces. Controls, as appropriate, were used and visualization was attained using the Envision Plus Detection System (Dako, Carpinteria, CA). Competition experiments were performed using recombinant proteins expressed in bacteria GST-Crx and the unrelated protein GST-Cry1 (Abnova H00001406-P01 and H00001407-P01) as well as Glutathione-S-Transferase (GST) alone (Millipore 12–350). Equimolar amounts of protein and anti-Crx antibody were incubated together for 30 minutes at room temperature and then applied to tissue sections as described above. Grading of immunoreactivity was based on the following semiquantitative approach by two neuropathologists (SS and KL): 0, no tumor cells demonstrating nuclear (for Crx) or membranous/cytoplasmic staining (for GFAP, Synaptophysin); 1+, <5% of tumor cells reactive; 2+, >5%–25% of tumor cells reactive; 3+, >25%–50% of tumor cells reactive; 4+, >50%–75% of tumor cells reactive; 5+, >75% of cells reactive.

**Table 1:** Antibody Panel Used In This Study.

Antigen	Clone	Dilution	Antigen Retrieval	Vendor
CRX	Polyclonal Rabbit (H-120)	1∶100	Citrate; Microwave	Santa Cruz Biotechnology, Santa Cruz, CA
OCT3/4(POU5F1)	Monoclonal Mouse (C-10; sc-5279)	1∶2000	Citrate, Steamer	Santa Cruz Biotechnology, Santa Cruz, CA
OLIG2	Polyclonal Rabbit (AB9610)	1∶15K	Citrate; pressure cooker	Millipore
GFAP	Polyclonal Rabbit (Z 0334)	1∶20K	Pressure cooker citrate	DAKO
Synaptophysin	Monoclonal Mouse (SY38)	1∶200	no treatment	DAKO

### Immunoblotting

50 ng and 20 ng of indicated proteins were spotted on nitrocellulose membranes. Blocking was performed for 1 hr using 5% BSA in PBS-T, followed by application of rabbit polyclonal anti-Crx antibody (H120; 1∶400 dilution in 0.1%BSA/PBS-T) for 30 min, 3 washes with PBS-T and incubation with anti-rabbit secondary antibody conjugated with HRP (1∶5000 dilution in 0.1%BSA/PBS-T). Following an additional three washes with PBS-T, the membrane was developed with chemiluminescent substrate (Thermo, SuperSignal West Pico 34078).

### *In Situ* Hybridization

Analysis of the expression pattern of Crx mRNA was conducted using GenePaint.org, an interactive publically available *in situ* hyrbidization (ISH) atlas of gene expression patterns in mouse embryos at developmental stage E14.5 (NMRI albino and C57BL/6 strains) and post-natal (P7) mouse brain (C57BL/6 strain). As described (http://www.genepaint.org/Frameset.html), gene expression was detected using digoxigenin-labeled antisense riboprobes generated by *in vitro* transcription from DNA templates and images and associated meta-data are deposited in a searchable public database (http://www.genepaint.org)[40], [41]. In situ hybridization studies for localization of CRX in post-natal mouse retina was performed according to standard protocols[40], [41]. Sequences for the DNA template used for in vitro transcription of the RNA probe were generated with primer sets available at http://www.genepaint.org.

### Animal Procedures

For immunohistochemistry studies, tissue specimens including brain and eye were harvested from E14.5 mouse pups that either had one (*Crx+/−*) or both (*Crx −/−*) alleles disrupted [39]. The specimens were snap frozen in liquid nitrogen and sections were prepared by cryostat sectioning. Genotyping was performed as previously described [39] and using probes designed by Transnetyx Inc.

### Animal Welfare Statement

All animals were handled in strict accordance with good animal practice as defined by the European Communities Council Directive of November 24, 1986 (86/609/EEC) and under authorization of Az 32.22/Vo (“Ordnungsamt der Stadt Göttingen”). All animals were euthanized for analysis using procedures approved by the appropriate institutional animal welfare committee.

### Expression Profiling Analysis

Expression profiling analysis of cell lines was performed using the publicly available Oncomine resource (http://www.oncomine.org/main/mianx,jsp) [42]and the publicly available datasets contained within the Oncomine database. Analysis was done using the t-test method for determining significance of *CRX* expression across multiple datasets for normal and cancer cell lines. All data was collected using U133 Plus 2.0 Affymetrix arrays. Global cancer cell line analysis was done using an unpublished but publicly available dataset created by Wooster et. al. in collaboration with GlaxoSmithKline which contained data from 316 cancer cell lines. Representative cell lines of pineal origin were not present in this dataset.

Meta-analysis of Crx expression was also assessed in a wide range of tumor types. Raw data files downloaded from public resources were first processed using the MAS5.0 algorithm implemented in Bioconductor to obtain detection calls and the 3′ to 5′ signal ratios for control probesets: GAPDH and β-ACTIN. All the CEL files were processed using the RMA algorithm implemented in Bioconductor to generate normalized expression values. Expression values were scaled by computing the median expression value for each sample and then processed using a custom script to scale the RMA-derived expression values such that each array will have the same median intensity. Expression profiling data from 1936 individual tissue samples and 929 individual cell line preparations were evaluated in this manner.

### Sequence Alignment

Sequence alignment (Smith-Waterman algorithm) of human CRX immunogen (amino acids 166 to 285) was performed with OTX1 and OTX2. The alignment conservation annotation is based on the AMAS method of multiple sequence alignment analysis[43]. The image was generated using Jalview 2.4.

## Results

### *Crx* mRNA Is Restricted to the Retina and Pineal

**Figure 1:**
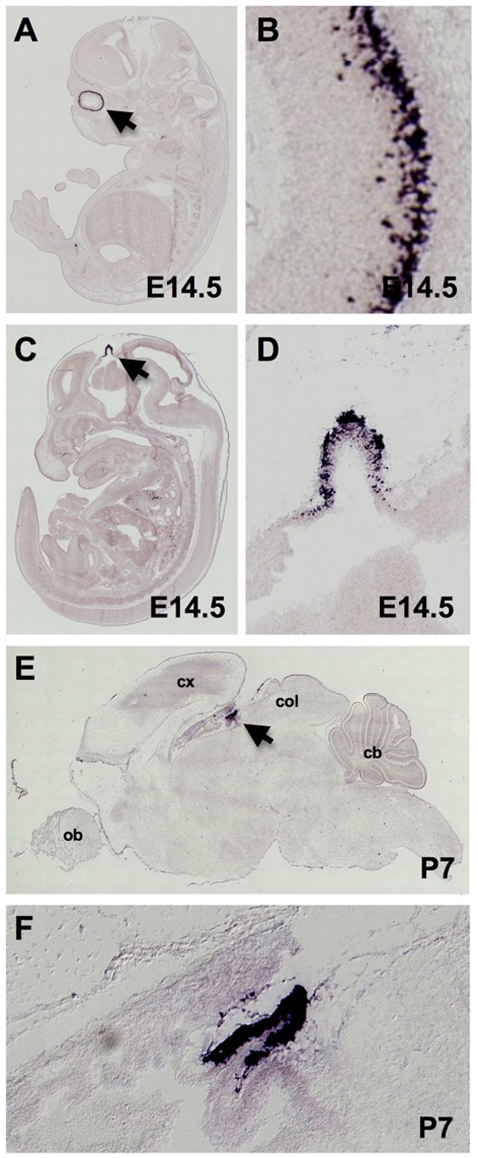
Strong expression of *Crx* mRNA is highly restricted to the retina and pineal during normal murine development. RNA *in situ* hybridization using antisense riboprobes for *Crx* at developmental stage E14.5 in NMRI mouse embryos (Genepaint Set ID DA117) demonstrates strong expression in the ventricular zone of the retina (A, B) and in the developing pineal which appears as a diverticulum in the diencephalic roof of the third ventricle (C, D). *Crx* in the brain of a P7 C57BL/6 mouse (Genepaint Set ID MH1082) demonstrates strong expression restricted to the pineal primordium (E, F)[40], [41].

To determine the degree of lineage restriction of Crx during development, we performed *in situ* hybridization on whole animal sections to detect *Crx* RNA at embryonic and postnatal stages. In 14.5 days post coitus NMRI mouse embryos (E14.5) *Crx* expression was restricted to the developing ventricular zone (VZ) progenitor cells of the retina and the pineal gland (Fig. 1A-D). At 7 postnatal days (P7), strong signal was detected in the outer nuclear layer of the retina with weak signal present in the inner nuclear layer (data not shown). In the brain of P7 mice, strong *Crx* RNA expression was mainly restricted to the pineal gland in C57BL/6 mice (Fig. 1E, 1F). Weak expression of Crx RNA was, however, also detected in the soft tissues of the face (Fig. 1A) and in a thin layer of the periventricular VZ progenitors of the developing posterior cerebral hemispheres (Fig. 1C). A similar pattern of expression to that seen in the NMRI embryo was also observed at stage E14.5 in C57BL/6 mouse embryos with a riboprobe recognizing a different portion of the Crx transcript (data not shown) in images obtained from the genepaint database (http://www.genepaint.org)[40], [41].

### Characterization of Anti-CRX Antibody H120

In light of the fact that CRX protein expression in situ has not been extensively analyzed, we sought to determine whether CRX expression might be similarly restricted in human at the protein level. To validate the specificity of the rabbit polyclonal antibody used in these studies (Table 1; Santa Cruz H120 anti-Crx) for recognition of Crx we took a combination of sequence alignment, immunoblotting and immunohistochemistry approaches.

**Figure 2:**
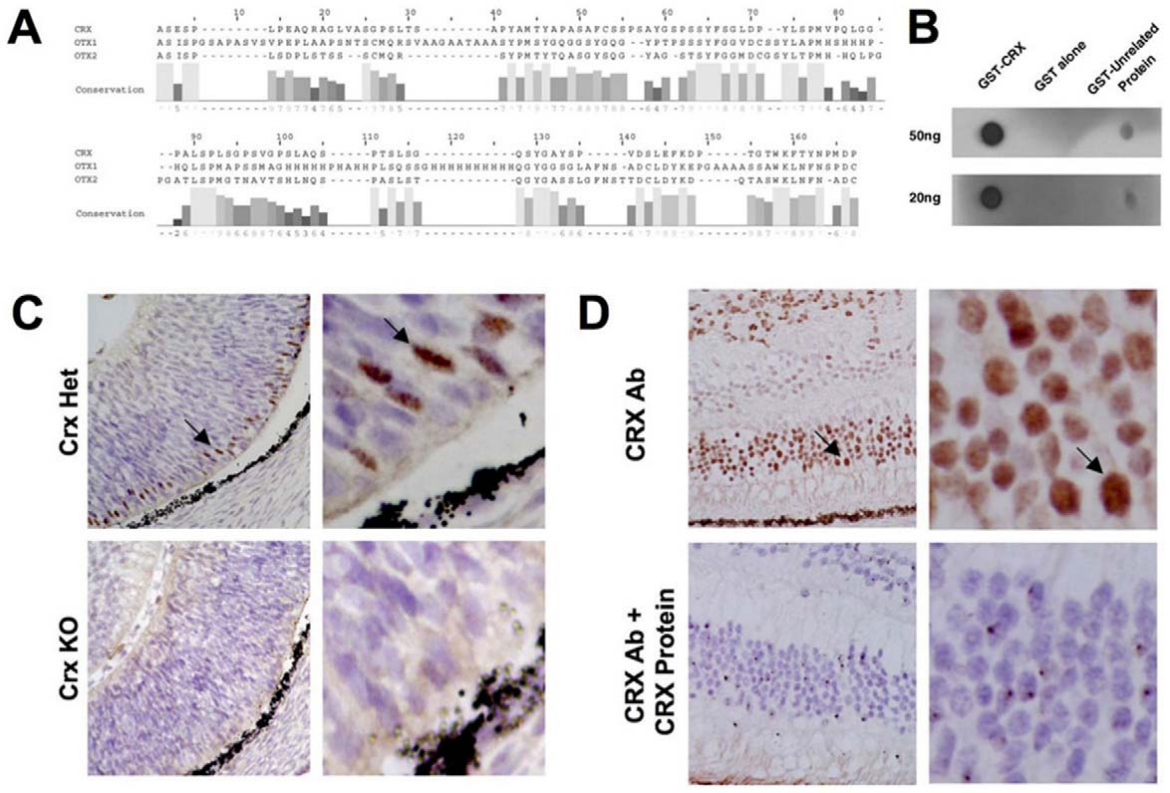
Validation of antibody specificity. Sequence alignment (Smith-Waterman) of human CRX immunogen (amino acids 166 to 285) with OTX1 and OTX2 (A) demonstrates low identity for the relevant epitope (<40%). Residue number 1 correpsonds with residue 166 of CRX. AMAS conservation scores are listed below the alignment. Immunoblot of native, non-denatured GST-Crx, GST alone and with the unrelated protein GST-Cry1 demonstrates robust specificity of the H120 antibody for native Crx (B). Immunohistochemical analysis using the H120 anti-Crx antibody demonstrates strong reactivity in the developing retina of E14.5 mice, which are heterozygous for Crx (*Crx* +/−), while the signal is absent in the retina of *Crx* knockout mice (*Crx* −/−) (C). Competition assays with CRX protein on tissue sections of human retina and retinoblastoma (cells at top of image) demonstrate the immunoreactivity of the H120 anti-Crx antibody is specifically competed away with 1∶1 molar amounts of purified GST-Crx protein in normal and tumor cells.

OTX1 and OTX2 are the proteins with the highest sequence identity to CRX in the genome of human and mouse. An alignment of the protein sequences in the region of human CRX that was used to generate the polyclonal antibody (amino acids 166 to 285) reveals that the overall identity between CRX and OTX1 (30%) and OTX2 (40%) is low reducing the likelihood of antibody cross reactivity (Fig. 2A). In fact in this region, homology is limited to stretches of three amino acids or less (with only one region of CRX and OTX1 sharing four amino acids), which is an insufficient length to likely support significant cross-reactivity of the antibody via a shared epitope.

We next performed immunoblots following SDS-PAGE resolution of lysates from human retina, human retinoblastoma cells and 293T cells with exogenously expressed Crx protein. Interestingly, we were unable to detect any band corresponding to Crx in any of these denatured lysates (data not shown). In fact no bands at all (background) were detected under these conditions. Incubation of the same Western blot with another antibody available through Santa Cruz (Q17 monoclonal) detected a band of the appropriate size. These findings led us to hypothesize that the H120 antibody was recognizing a conformational epitope rather than a linear epitope, a relatively frequent event according to published studies[44]. To test this we performed dot blots of native (non-denatured) GST-CRX protein purified from bacteria and were successfully able to detect the native protein with the H120 Crx antibody while we were unable to detect control unrelated proteins or GST alone (Fig. 2B).

Since the H120 anti-Crx antibody recognizes a conformational rather than a linear epitope, we further characterized the specificity of the antibody in the context of *in vivo* staining. We demonstrate that the H120 anti-Crx antibody recognizes a strong signal in appropriate regions of the developing retina of E14.5 mice that are heterozygous for Crx (Crx +/−) but that the signal is completely absent in the retina of Crx knockout mice (Crx −/−) which lack only the Crx protein through homologous recombination [39](Fig. 2C). In addition, the pattern of immunoreactivity detected in the mouse CNS using the H120 anti-CRX antibody (retina and pineal) mirrors the pattern of CRX mRNA expression and not that of OTX1 and OTX2 mRNA expression as determined by *in situ* hybdridization (Fig. S1). To further address the specificity of the antibody in the context of human tissue, we performed competition assays with purified CRX protein and show on tissue sections of human retinoblastoma and adjacent uninvolved retina that the immunostaining with the H120 anti-Crx antibody can be completely competed away with 1∶1 molar amounts of purified GST-Crx protein in both normal retina and the retinoblastoma tumor cells (Fig. 2D).

### CRX Protein Is Highly Expressed in Human Retina and Pineal

**Figure 3:**
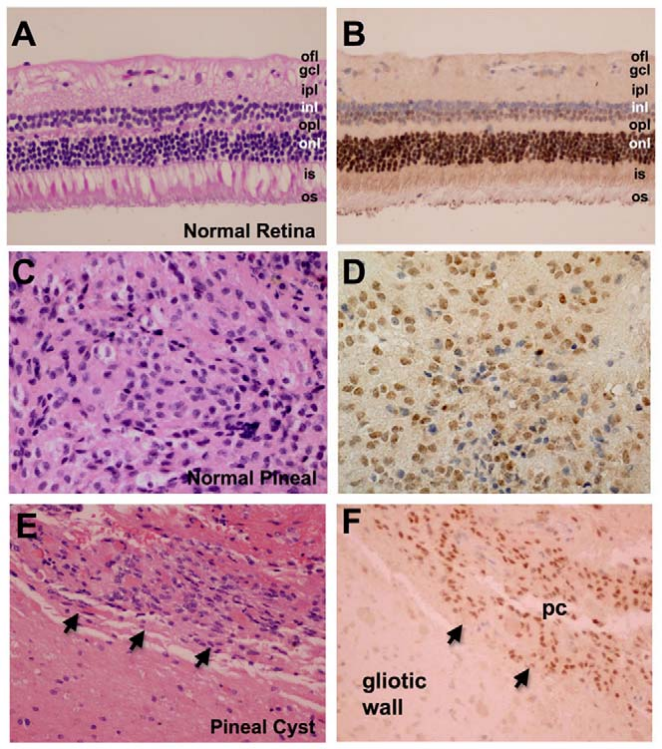
CRX protein is highly expressed in the normal adult human retina and pineal. Immunohistochemical analysis of CRX expression demonstrates robust expression in the nuclei of cones and rod cells in the outer nuclear layer (onl) as well as much weaker expression in a subset of nuclei in the inner nuclear layer (inl) that abut the outer plexiform layer (opl) of the adult human retina (A, B). This region is populated predominantly by horizontal and bipolar cells. The ganglion cell layer does not express CRX. Expression of CRX in the pinealocytes of a surgically resected normal pineal gland (C, D) and pineal cyst (E, F arrows). Gliotic regions composing the cyst wall do not express CRX (lower left corner of E, F). Optic fiber layer (ofl), ganglion cell layer (gcl), inner plexiform layer (ipl), inner segment (is), outer segment (os), pineal cyst (pc). Original magnification 200x (A, B, E, F) and 400x (C, D).

Having characterized the specificity of the antibody for recognition of Crx, we turned to evaluating the pattern of expression of CRX in the human eye and pineal. Analysis of normal appearing adult human retina (Fig. 3A,3B) from eye enucleation specimens showed robust expression of CRX protein in 95% of cases (19/20). Expression was detected in nearly all of the nuclei of cone and rod photoreceptor cells in the outer nuclear layer (Fig. 3B). Weaker expression was demonstrated in the nuclei of the inner nuclear layer (Fig. 3B) in the cells immediately adjacent to the outer plexiform layer of the retina, consistent with previously reported results[30], [45]–[47]. This region of the inner nuclear layer is populated predominantly by the nuclei of bipolar and horizontal cells. Intranuclear staining was absent in ganglion cells (Fig. 3B).

Expression of CRX protein in the human pineal was also evaluated by immunohistochemistry in eight pineal surgical resection specimens that contained either normal pineal (n = 5) (Fig. 2C) or a pineal cyst (n = 3) (Fig. 3E). In all eight specimens intranuclear staining was present in >85% of pineocyte nuclei (Fig. 3D, 3F). Gliotic regions composing the cyst wall were negative for intranuclear CRX immunostaining (Fig. 3F) as were inflammatory cells and endothelial and smooth muscle cells composing blood vessels. To note, expression of CRX was detected by immunohistochemistry in none of the ten post-mortem pineal specimens evaluated. The presence of CRX in surgically-derived pineal specimens but the absence of CRX immunostaining in autopsy-derived tissue likely reflects the labile nature of the antigen and highlights the importance of performing CRX immunohistochemistry on recent resection specimens that have undergone prompt formalin-fixation after resection.

### CRX Is a Sensitive Marker for Retinoblastoma and Pineal Parenchymal Tumors

**Figure 4:**
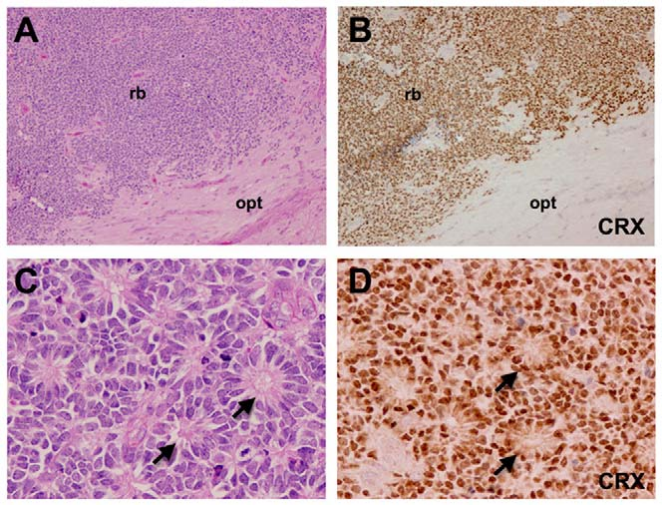
CRX is a sensitive marker for retinoblastoma. Human enucleation specimen with retinoblastoma (rb) abutting and infiltrating a portion of optic nerve (A, opt), immunohistochemical staining for CRX demonstrates robust expression in the nuclei of most all tumor cells and an absence of CRX expression in cells of the optic nerve (B). 100x original magnification. A higher magnification view of human retinoblastoma demonstrating distinct Flexner-Wintersteiner rosettes (C, D arrows) also reveals strong expression of CRX in tumor nuclei particularly in rosettes (D). Original magnification 400x.

CRX protein expression was evaluated by immunohistochemistry in 22 enucleation specimens for histologically confirmed retinoblastoma (Fig 4A, 4C). 21 of the 22 cases (>95%) demonstrated strong intranuclear CRX immunoreactivity in most tumor cells (Fig 4B, 4D) with strong staining evident in both well-differentiated regions demonstrating Flexner-Wintersteiner rosettes (Fig. 4D) as well as in moderately and poorly differentiated regions (Fig. 4B). CRX immunostaining was negative in the regions of optic nerve adjacent to the retinoblastoma (Fig. 4B, lower right portion of field). CRX immunostaining was absent in necrotic regions and often in morphologically viable cells surrounding these necrotic regions. In addition, we noted CRX expression was weak or absent in the central portion of large tumors while the peripheral portions and the associated retina demonstrated strong expression. These findings, in addition to the absence of CRX staining in post-mortem pineal tissue, suggest that the CRX antigen is moderately labile and needs to be evaluated in well-fixed tissue.

**Figure 5:**
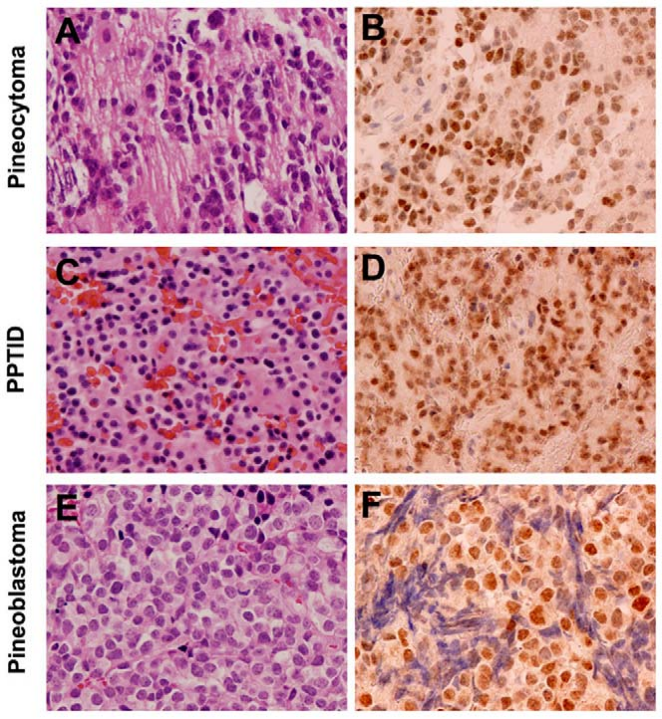
CRX is a sensitive marker for tumors of pineal lineage. Immunohistochemistry reveals robust intranuclear expression of CRX in the nuclei of various tumors of pineal lineage: pineocytoma (A, B), pineal parenchymal tumor of intermediate differentiation (C, D) and a pineoblastoma (E, F). Original magnification, 400x.

CRX expression was also evaluated by immunohistochemistry in 13 pineal parenchymal tumors that were classified according to WHO criteria. Included among these were four pineocytoma (W.H.O. Grade I) (Fig. 5A), four pineal parenchymal tumors of intermediate differentiation (W.H.O. Grade II/III) (Fig. 5C), and five pineoblastoma (W.H.O. Grade IV) (Fig. 5E). These tumors had previously solely been evaluated with GFAP and synaptophysin to arrive at a clinical diagnosis (Table 2). Twelve of the 13 cases demonstrated intranuclear staining for CRX (4 of 4 pineocytoma, Fig. 5B; 4 of 4 pineal parenchymal tumor of intermediate differentiation, Fig. 5D; and 4 of 5 pineoblastoma Fig. 5F). Four of the five pineoblastoma demonstrated intranuclear staining in >50% of tumor cells, 3 of the 4 pineal parenchymal tumors of intermediate differentiation demonstrated intranuclear staining in 50% of tumor cells and 3 of the 4 pineocytoma demonstrated intranuclear staining in >50% of the tumor cells (Table 2). The heterogeneity of CRX staining in a portion of the tumor samples may reflect biological heterogeneity within these tumors. Of these pineal parenchymal tumors, 12 of 13 demonstrated immunoreactivity for synaptophysin, currently the most widely used marker of pineal tumors, in >75% of tumor cells and 12 of 13 demonstrated immunoreactivity for GFAP, a glial marker, in <5% of tumor cells. Examination of serial sections showed that the CRX staining correlated precisely to regions of synaptophysin signal in a highly specific manner in 100% of cases. In all, the data suggest that CRX is a sensitive diagnostic marker for tumors of pineal lineage and can be used effectively along with synaptophysin and GFAP in the diagnostic evaluation of these tumors.

**Table 2:** Immunohistochemical Staining Results on Pineal Parenchymal Tumors.

Case Number	Tumor	Age (years)	Gender	CRX	GFAP	Synaptophysin
1	Pineocytoma	71	Female	2+	0	5+
2	Pineocytoma	56	Female	4+	0	4+
3	Pineocytoma	61	Male	4+	0	4+
4	Pineocytoma	54	Male	4+	3+	2+
5	PPTID	2	Male	4+	0	4+
6	PPTID	30	Male	2+	0	4+
7	PPTID	44	Female	4+	0	5+
8	PPTID	36	Female	4+	0	4+
9	Pineoblastoma	4	Male	4+	1+	5+
10	Pineoblastoma	12	Female	4+	0	5+
11	Pineoblastoma	13	Male	5+	0	5+
12	Pineoblastoma	9	Male	5+	1+	5+
13	Pineoblastoma	8	Female	0	0	4+

Table 2 Legend:

0 indicates no staining; 1+, <5% tumor cells reactive; 2+, 5% to 25% tumor cells reactive; 3+, 26% to 50% tumor cells reactive; 4+, 51% to 75% tumor cells.

reactive; 5+, >75% tumor cells reactive. GFAP, Glial Fibrillary Acid Protein; Pineal Parenchymal Tumor of Intermediate Differentiation (PPTID).

### CRX Is a Specific Marker for Tumors of Pineal/Retinal Lineages

**Figure 6:**
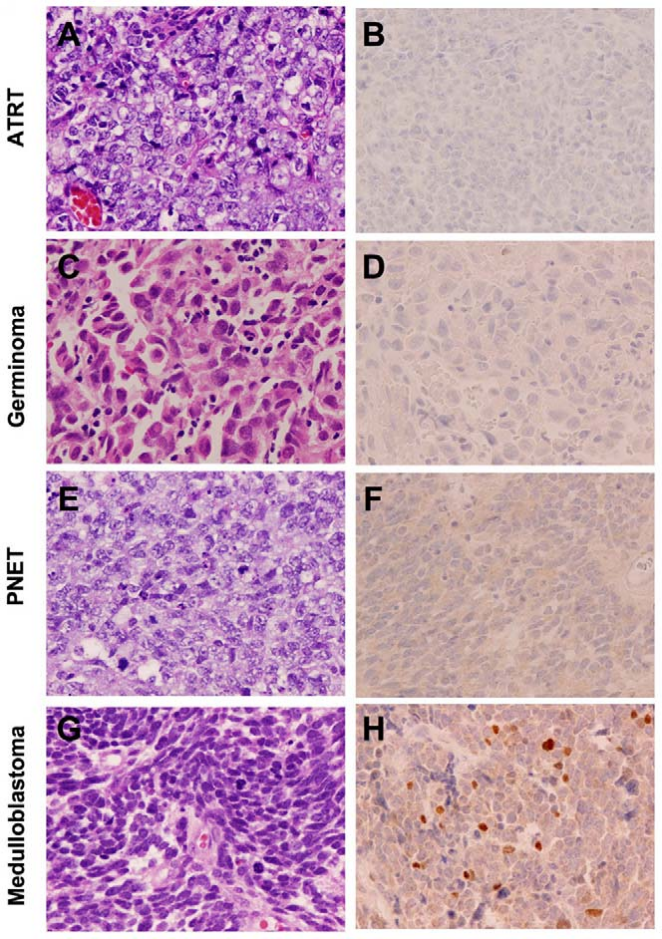
CRX is a specific marker for tumors of retinal and pineal lineage. Immunohistochemistry for CRX in tumors frequently considered in the differential diagnosis of pineoblastoma including atypical teratoid rhabdoid tumor (ATRT) (A, B), CNS germinoma (C, D) and primitive neuroectodermal tumor (PNET) (E, F) reveals an absence of staining. Immunostaining for CRX in classic medulloblastoma reveals heterogeneous intranuclear staining in scattered cells in a subset of tumors (G, H). Original magnification, 400x.

The diagnosis of tumors of the pineal region is often difficult due to the range of tumor types that can affect this region and the often minute size of the biopsy that is provided for definitive diagnosis. To investigate the specificity of CRX in the diagnosis of tumors of pineal/retinal lineage, we performed immunohistochemistry for CRX on a number of tumor types that frequently enter the differential diagnosis of pineal masses (Fig. 6A–6F). Intranuclear immunoreactivity was not detected in a broad range of tumors examined including five atypical teratoid/rhabdoid tumors (Fig. 6A, 6B), nine germinoma (Fig. 6C, 6D), five primitive neuroectodermal tumors (Fig. 6E, 6F), four embryonal carcinoma, five choroid plexus carcinoma, six anaplastic ependymoma, five metastatic carcinoma, five neurocytoma, five Langerhans cell histiocytosis, five meningioma and 24 high-grade diffuse gliomas (12 glioblastoma and 12 anaplastic oligodendrogliomas).

Pathologic distinction of medulloblastoma from pineoblastoma can occasionally pose a clinical challenge given that these poorly differentiated tumors are morphologically indistinguishable and larger tumors may grow to involve both the superior cerebellum and pineal regions. Interestingly, four of ten medulloblastoma (Fig. 6G) demonstrated a subpopulation of scattered cells with positive intranuclear CRX staining (Fig. 6H). Two of the cases showed immunoreactivity in <5% of the tumor cells, one case in 5–25% of tumor cells and one in 25–50% of tumor cells. While this heterogeneous pattern of immunoreactivity is noteworthy, none of the cases demonstrated the robust, uniform pattern of CRX immunostaining most often seen in pineoblastoma.

**Figure 7:**
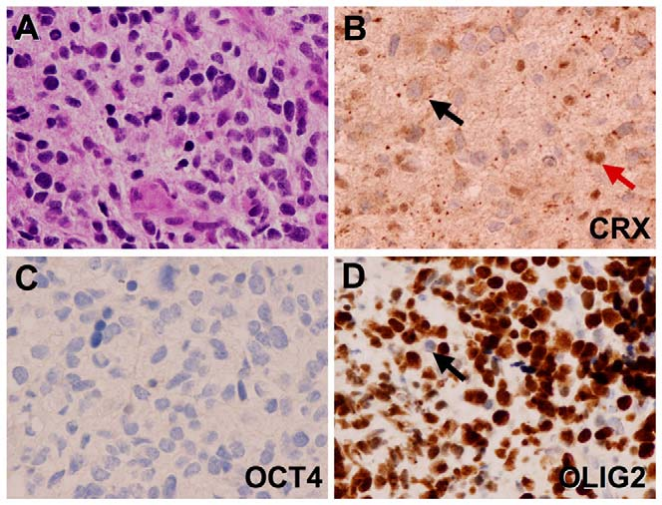
Diagnostic use of lineage-specific transcription factor “code” in evaluation of a glioblastoma of the pineal region. Examination of H&E stained sections of a pineal tumor reveals high-grade histologic features somewhat suggestive of glioblastoma (A). Immunohistochemistry showed nuclear CRX in normal pineocytes (red arrow) that are infiltrated by the invasive tumor which is negative for CRX (black arrow) (B). The germ cell marker OCT4 is absent in neoplastic cells and in pineocytes (C). However, robust expression of OLIG2 is evident in neoplastic cells but absent in infiltrated pineocytes (arrow) (D). Original magnification 400x.

A specific example of the practical diagnostic use of CRX immunohistochemistry can be provided by a recent case evaluated at Children's Hospital, Boston of a high-grade neoplasm of the pineal region (Fig. 7A). This tumor represented the frequent diagnostic challenge presented by tumors of the pineal. This particular tumor was identified as a high-grade neoplasm of uncertain origin based on a minute surgical biopsy specimen. A panel of lineage specific transcription factors including CRX (pineal), OLIG2 (diffuse gliomas) and OCT4 (germ cell tumors) were utilized to “decode” the lineage of the tumor. In this case CRX and the germ cell transcription factor OCT4 were negative and showed no intranuclear reactivity in tumor cells (Fig. 7B and 7C), while OLIG2 exhibited strong nuclear staining (Fig. 7D) consistent with a high-grade glioma arising principally in the pineal region.

As seen in Figure 7B, we also found that a low percentage of tumors demonstrated weak granular cytoplasmic immunoreactivity for CRX. This pattern of cytoplasmic staining was particularly seen in gliomas, Langerhans cell histiocytosis, central neurocytoma, primitive neuroectodermal tumor and renal cell carcinoma. In addition, the cytoplasm of lymphocytes demonstrated weak to moderate immunoreactivity as noted in tumors bearing many of these cells such as some germinoma. The cytoplasmic pattern of staining appears to be consistent with cross-reactivity within the endoplasmic reticulum and/or secretory granules and was not considered to be specific staining.

**Figure 8:**
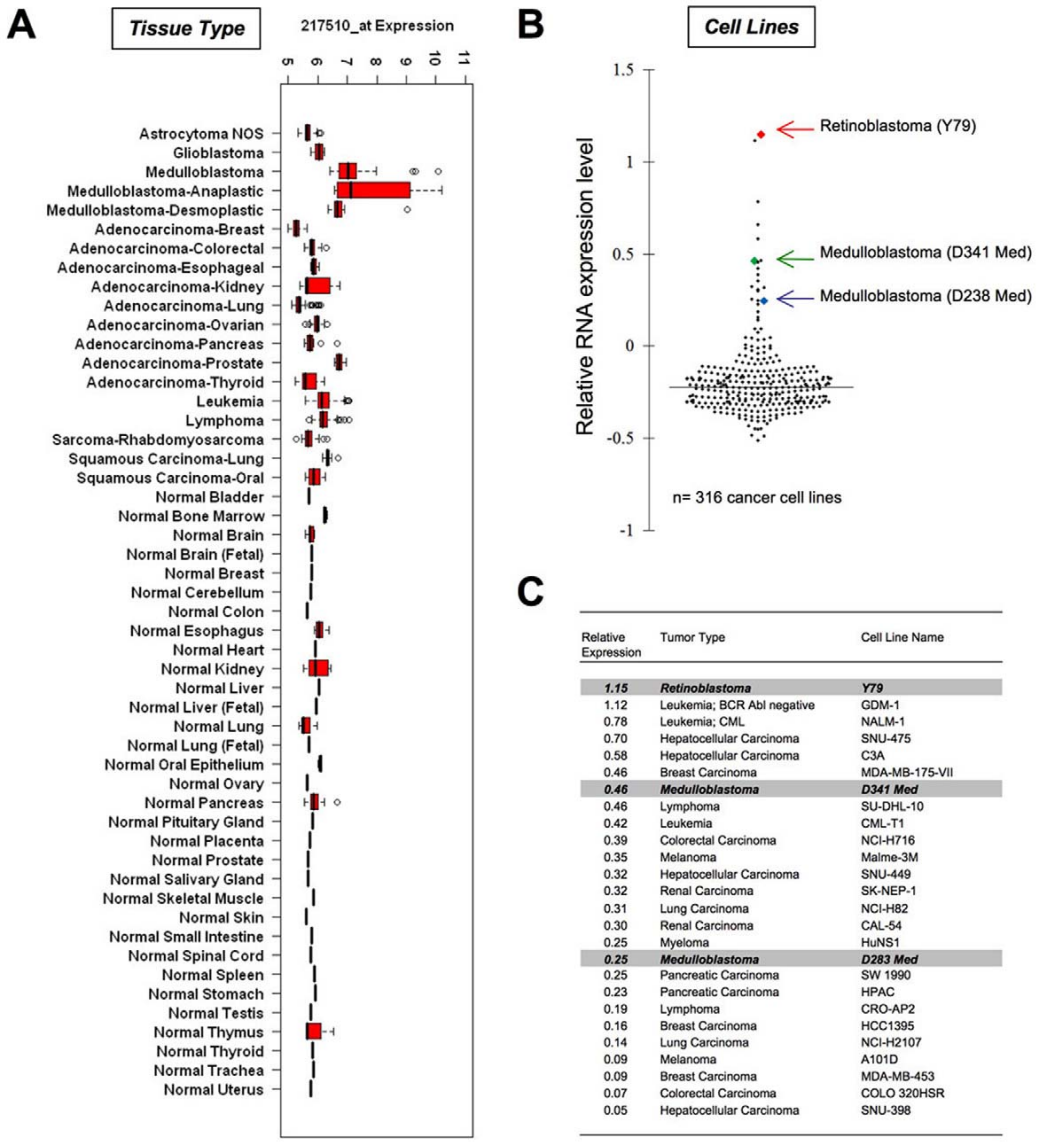
Expression profiling analysis demonstrates *CRX* expression is highly lineage-restricted across a broad range of cancer tissues and cell lines. Analysis of *CRX* mRNA expression was assessed using publically available expression profiling data from over 1900 primary tumor samples and demonstrates elevated expression of *CRX* predominantly in medulloblastoma samples (A). Pineal parenchymal tumor data is not available. Oncomine data from 316 human cancer cell lines demonstrates 26 lines with significant expression of *CRX* relative to other lines as demonstrated in a scatter plot (B). All expression levels are relative to the total dataset. The highest relative expression level was present in the sole retinoblastoma cell line (Y79) within the dataset (C). High level expression was also noted in the only two medulloblastoma cell lines present in the dataset (D341 and D283). Other CNS tumor cell lines showed no significant increase in *CRX* expression (8 astrocytoma, 1 PNET). Additional validation using same independent normalization methods as for tissues produced similar results (Fig. S2). Data utilized in construction of plots is provided as Table S1 and S2.

Finally, to more broadly investigate the extent of *CRX* expression across many types of cancer and normal tissues, we performed a meta-analysis of expression profiling data from a wide range of cancer tissues, normal tissue controls and cancer cells lines using a wide sampling of publicly available datasets (Table S1). Meta-analysis of 1,934 primary tumor and normal tissue samples of various types revealed high levels of Crx expression was restricted to a subset of medulloblastoma specimens, particularly those of anaplastic subtype (Fig. 8A). Publicly available datasets from retinoblastoma or pineoblastoma were not identified in this analysis. Furthermore, analysis of a comprehensive panel of 318 different human cancer cell lines (Wooster et al.; Oncomine database of transcriptome profiles (http://www.oncomine.org)[42], [48], [49]) using both Oncomine analysis methods (Figure 8B,C), as well as the normalization methods used in tumor tissues above (Fig. S2) demonstrated that the highest relative level of *CRX* expression was present in the sole retinoblastoma cell line (Y79) present in the dataset (Fig. 8B and 8C) and that moderate levels of *CRX* expression were also noted in two medulloblastoma cell lines (D341-Med and D283-Med) and a handful of other non-CNS tumor cell lines. Overall, the vast majority of tumor lines showed no evidence of consistent expression of *CRX*, including 8 glioma cell lines and 1 central PNET cell line. No expression profiles of tumors or cell lines of pineal parenchymal origin were available for comparison within public databases.

## Discussion

Advances in the study of the normal pineal and pineal region tumors has been limited in part due to their very infrequent occurrence, with tumors of the pineal region accounting for less than 0.1% of all intracranial tumors. Here we hypothesized that lineage restricted transcription factors for retino-pineal progenitors might represent useful diagnostic and investigational tools as has been demonstrated in other cancers[28], [29], [50], [51]. The molecular-genetic similarity between the retina and pineal and the remarkably restricted expression pattern of *Crx* mRNA suggested a distinct opportunity for employing *Crx* as a candidate biomarker. Our studies of RNA ISH in whole embryos and brain confirm the remarkable lineage restiction of this gene across the whole mouse embryo. Furthermore, our studies in human systems using IHC and expression profiling data validate that such lineage restriction is highly preserved in humans as well. Given that CRX protein expression had not been previously as well studied, we find that the RNA and protein expression are highly conserved with no significant differences detected in our study.

From a diagnostic standpoint, we find that >90% of retinoblastoma and >90% of pineal parenchymal tumors display significant intranuclear expression of CRX while none of the tumors entering the differential diagnosis of pineal masses display intranuclear CRX immunoreactivity. These findings highlight that CRX is both a sensitive and specific marker for tumors of pineal and retinal lineage and that its use should be further evaluated for routine application as an essential component of the standard workup of tumors of the pineal region. Previous studies using non-TF markers of photoreceptor lineage have also supported the lineage conservation between tumors of these two regions, but due to their non-nuclear and less consistent expression have found little diagnostic acceptance in clinical practice[20], [52]. These same studies in the retina had concluded that retinoblastomas represented a bias towards cone differentiation, and the presence of strong CRX staining supports this given its more specific role in development of cone photoreceptors[52]. Also, while immunohistochemistry for CRX may be of practical utility in the classification of biopsies of the central nervous system it may also be valuable in unequivocally ascribing peripheral metastases in bone marrow and elsewhere to a known primary ocular retinoblastoma as well as in the evaluation of cerebrospinal fluid cytological specimens in patients with retinoblastoma, pineoblastoma and pineal parenchyal tumor of intermediate differentiation. Finally we find that CRX is a new sensitive and specific marker for retinoblastoma and pineal parenchymal tumors that should be useful in the diagnostic evaluation of pineal masses when used as part of a panel of immunohistochemical markers including synaptophysin and GFAP.

An interesting finding in this study is that CRX is expressed in a heterogeneous subpopulation of cells in four out of the ten medulloblastomas that were analyzed. Photoreceptor differentiation has previously been demonstrated in medulloblastoma with retinal S-antigen and rhodopsin antigens detected by immunohistochemistry[53]–[55]. A recent study classifying medulloblastoma based on gene expression profiles identified five molecular-genetic subtypes, two of which demonstrated photoreceptor differentiation[56]. These subtypes represented approximately 40% of medulloblastoma cases, similar to our findings, and had increased RNA expression of the photoreceptor transcription factors *CRX*, *NRL* and *NR2E3*. In addition, they were associated with clinical presentation at a younger age (<3 years of age) and more aggressive biological behavior with an increased risk of metastases at the time of diagnosis. These findings support the pathologic and molecular heterogeneity of medulloblastoma [57], [58] and suggest that in addition to a role in the diagnosis of pineal and retinal tumors, CRX immunohistochemistry may provide critical information in determining subtype classification and poor prognosis in cases of medulloblastoma [59].

Lineage-specific transcription factors have increasingly been demonstrated as important tools in the diagnostic workup of a range of tumor types including OCT4 and NANOG in peripheral and CNS germ cell tumors[28], [29], MYF4 in tumors with myogenic differentiation[60], OLIG2 in tumors with glial differentiation[61], TTF1 in thyroid tumors, BSAP (PAX5) in B-cell neoplasms[62], Brachyury in chordomas [63] and hemangioblastomas [64] and CDX2 in gastrointestinal tumors[65]. CRX represents a new addition to this group and suggests that additional useful markers may be discovered through systematic identification of lineage restricted transcription factors in a broader range of tumors.

Evidence from Fevre-Montange et al. demonstrates that at least seven genes are specifically expressed in pineoblastoma versus other tumors of pineal parenchymal origin. Consistent with our results showing that the frequency and intensity of expression of CRX protein is similar in pineoblastoma, PPTID and pineocytoma, *CRX* was not among the list of genes which discriminated classes of pineal parenchymal tumors. Among the seven discriminatory genes from Fevere-Montange et al., were the three transcription factors *HOXD13*, *PITX2* and *POU4F2* [*25*], which unlike the highly lineage-restricted pattern of expression seen for *Crx*, are expressed in lineage-nonrestricted patterns. The use in tandem of both lineage-restricted and lineage-nonrestricted transcription factors as components of a diagnostic panel has been used in the evaluation of germ cells tumors where lineage-restricted transcription factors like OCT4 and NANOG can be used alongside lineage-nonrestricted transcription factors like SOX2 [29] and SOX17[66]. This panel of transcription factors permits, first, the confident identification of a germ cell tumor and second, the more subtle discrimination between germ cell tumor subclasses such as seminoma/germinoma, embryonal carcinoma, yolk sac tumor and choriocarcinoma. The findings of Fevre-Montagne et al. suggest that CRX along with HOXD13, PITX2 and POU4F2 might form the core transcription factor code permitting an objective immunohistochemical and molecular subclassification of pineal parenchymal tumors.

Within the field of cancer research, extensive effort continues to be directed at identifying oncogene pathways that are activated in cancer with the goal of developing targeted therapeutics to specific signaling pathways. An emerging body of evidence, however, supports the concept that tumors may also be dependent on the same lineage-specific transcription factors which critically regulate the normal tissue restricted developmental progenitor cells[50], [51], [67]. This dependence for survival and proliferation on critical cellular constituents that are not mutated and that alone do not serve to transform cells (sometimes called ‘non-oncogene addiction’) represents an under-explored opportunity for development of targeted cancer therapies directed at these components and pathways. Such pathways also have the added benefit of reduced off-target effects due to their inherent lineage restriction. Our study suggests CRX might represent just such a target in retinoblastoma, pineoblastoma, and possibly even a subset of medulloblastoma. Functional studies of CRX in these cancers would certainly seem warranted, given the known dependency of retinal and pineal lineage cells on this factor in humans and mice. While no evidence of CRX alterations in cancer have yet been described, its expression at high levels in these tumors also warrants further investigation as to whether it might be a target for rearrangement or amplification in cancers. Review of our expression profiling data on cancer cell lines suggests that its expression may be aberrantly regulated in several other cancers not of the CNS lineage. Our identification of retinoblastoma and medulloblastoma cell lines with *CRX* expression also represents an opportunity for performance of further functional studies of CRX in these specific human cell line systems given the lack of readily available model systems for these cancers.

## Supporting Information

Figure S1 Comparison of H120 antibody staining (Crx) with RNA expression pattern of Otx1 and Otx2. Immunohistochemistry for Crx using H120 antibody in E14.5 mouse (A, B, C, brown DAB) shows no overlap with the RNA expression pattern (purple, BMPurple) of Otx1 (D, E, F) or Otx2 (G, H, I), including areas with high level Otx expression such as the olfactory bulb and cerebellum. This suggests that H120 is specific for Crx and does not recognize the closest predicted family members.
(2.18 MB TIF)

Figure S2 Meta-analysis of expression profiling in cancer cell lines shows high level expression of CRX in retinoblastoma and medulloblastoma cell lines. The Glaxo-Smith-Kline human cancer cell line dataset was normalized and analyzed for expression of the CRX specific probeset 217510. Highest expression was seen in two medulloblastoma cell lines and the single retinoblastoma cell line in the dataset. Most other cell lines showed little to no expression of CRX.
(2.52 MB TIF)

Table S1 CRX Expression in Human Tissues
(0.23 MB XLS)

Table S2 CRX Expression in Human Cell Lines
(0.17 MB XLS)

## References

[1] Ironside JW, Moss TH, Louis DN, Lowe JS, Weller RO (2001). Diagnostic Pathology of Nervous System Tumours: Churchill Livingstone..

[2] De Girolami U, Fevre-Montange M, Seilhean D, Jouvet A (2008). Pathology of tumors of the pineal region.. Rev Neurol (Paris).

[3] Hirato J, Nakazato Y (2001). Pathology of pineal region tumors.. J Neurooncol.

[4] Jouvet A, Saint-Pierre G, Fauchon F, Privat K, Bouffet E (2000). Pineal parenchymal tumors: a correlation of histological features with prognosis in 66 cases.. Brain Pathol.

[5] Louis DN, Ohgaki H, Wiestler OD, Cavenee WK, Burger PC (2007). The 2007 WHO classification of tumours of the central nervous system.. Acta Neuropathol.

[6] Scheithauer BW (1999). Pathobiology of the pineal gland with emphasis on parenchymal tumors.. Brain Tumor Pathol.

[7] Scheithauer BW, Fuller GN, VandenBerg SR (2008). The 2007 WHO classification of tumors of the nervous system: controversies in surgical neuropathology.. Brain Pathol.

[8] Gilheeney SW, Saad A, Chi S, Turner C, Ullrich NJ (2008). Outcome of pediatric pineoblastoma after surgery, radiation and chemotherapy.. J Neurooncol.

[9] Parwani AV, Baisden BL, Erozan YS, Burger PC, Ali SZ (2005). Pineal gland lesions: a cytopathologic study of 20 specimens.. Cancer.

[10] Vogel H, Fuller GN (2003). Primitive neuroectodermal tumors, embryonal tumors, and other small cell and poorly differentiated malignant neoplasms of the central and peripheral nervous systems.. Ann Diagn Pathol.

[11] Min KW, Seo IS, Song J (1987). Postnatal evolution of the human pineal gland. An immunohistochemical study.. Lab Invest.

[12] Hassoun J, Gambarelli D, Peragut JC, Toga M (1983). Specific ultrastructural markers of human pinealomas. A study of four cases.. Acta Neuropathol.

[13] Herrick MK, Rubinstein LJ (1979). The cytological differentiating potential of pineal parenchymal neoplasms (true pinealomas). A clinicopathological study of 28 tumours.. Brain.

[14] Jouvet A, Fevre-Montange M, Besancon R, Derrington E, Saint-Pierre G (1994). Structural and ultrastructural characteristics of human pineal gland, and pineal parenchymal tumors.. Acta Neuropathol.

[15] Nielsen SL, Wilson CB (1975). Ultrastructure of a “pineocytoma”.. J Neuropathol Exp Neurol.

[16] Korf HW, Klein DC, Zigler JS, Gery I, Schachenmayr W (1986). S-antigen-like immunoreactivity in a human pineocytoma.. Acta Neuropathol.

[17] Perentes E, Rubinstein LJ, Herman MM, Donoso LA (1986). S-antigen immunoreactivity in human pineal glands and pineal parenchymal tumors. A monoclonal antibody study.. Acta Neuropathol.

[18] Mena H, Rushing EJ, Ribas JL, Delahunt B, McCarthy WF (1995). Tumors of pineal parenchymal cells: a correlation of histological features, including nucleolar organizer regions, with survival in 35 cases.. Hum Pathol.

[19] van Veen T, Ostholm T, Gierschik P, Spiegel A, Somers R (1986). alpha-Transducin immunoreactivity in retinae and sensory pineal organs of adult vertebrates.. Proc Natl Acad Sci U S A.

[20] Lopes MB, Gonzalez-Fernandez F, Scheithauer BW, VandenBerg SR (1993). Differential expression of retinal proteins in a pineal parenchymal tumor.. J Neuropathol Exp Neurol.

[21] Sawai J, Nakazato Y, Yamane Y, Kimura N, Kishi S (2003). Immunohistochemical localization of human pineal tissue antigens in normal retina and retinoblastomas.. Neuropathology.

[22] Amoaku WM, Willshaw HE, Parkes SE, Shah KJ, Mann JR (1996). Trilateral retinoblastoma. A report of five patients.. Cancer.

[23] De Potter P, Shields CL, Shields JA (1994). Clinical variations of trilateral retinoblastoma: a report of 13 cases.. J Pediatr Ophthalmol Strabismus.

[24] Kivela T (1999). Trilateral retinoblastoma: a meta-analysis of hereditary retinoblastoma associated with primary ectopic intracranial retinoblastoma.. J Clin Oncol.

[25] Fevre-Montange M, Champier J, Szathmari A, Wierinckx A, Mottolese C (2006). Microarray analysis reveals differential gene expression patterns in tumors of the pineal region.. J Neuropathol Exp Neurol.

[26] Hattab EM, Tu PH, Wilson JD, Cheng L (2005). OCT4 immunohistochemistry is superior to placental alkaline phosphatase (PLAP) in the diagnosis of central nervous system germinoma.. Am J Surg Pathol.

[27] Ngan KW, Jung SM, Lee LY, Chuang WY, Yeh CJ (2008). Immunohistochemical expression of OCT4 in primary central nervous system germ cell tumours.. J Clin Neurosci.

[28] Santagata S, Hornick JL, Ligon KL (2006). Comparative analysis of germ cell transcription factors in CNS germinoma reveals diagnostic utility of NANOG.. Am J Surg Pathol.

[29] Santagata S, Ligon KL, Hornick JL (2007). Embryonic stem cell transcription factor signatures in the diagnosis of primary and metastatic germ cell tumors.. Am J Surg Pathol.

[30] Hsiau TH, Diaconu C, Myers CA, Lee J, Cepko CL (2007). The cis-regulatory logic of the mammalian photoreceptor transcriptional network.. PLoS ONE.

[31] Gamse JT, Shen YC, Thisse C, Thisse B, Raymond PA (2002). Otx5 regulates genes that show circadian expression in the zebrafish pineal complex.. Nat Genet.

[32] Li X, Chen S, Wang Q, Zack DJ, Snyder SH (1998). A pineal regulatory element (PIRE) mediates transactivation by the pineal/retina-specific transcription factor CRX.. Proc Natl Acad Sci U S A.

[33] Nishida A, Furukawa A, Koike C, Tano Y, Aizawa S (2003). Otx2 homeobox gene controls retinal photoreceptor cell fate and pineal gland development.. Nat Neurosci.

[34] Freund CL, Gregory-Evans CY, Furukawa T, Papaioannou M, Looser J (1997). Cone-rod dystrophy due to mutations in a novel photoreceptor-specific homeobox gene (CRX) essential for maintenance of the photoreceptor.. Cell.

[35] Jacobson SG, Cideciyan AV, Huang Y, Hanna DB, Freund CL (1998). Retinal degenerations with truncation mutations in the cone-rod homeobox (CRX) gene.. Invest Ophthalmol Vis Sci.

[36] Sohocki MM, Bowne SJ, Sullivan LS, Blackshaw S, Cepko CL (2000). Mutations in a new photoreceptor-pineal gene on 17p cause Leber congenital amaurosis.. Nat Genet.

[37] Swain PK, Chen S, Wang QL, Affatigato LM, Coats CL (1997). Mutations in the cone-rod homeobox gene are associated with the cone-rod dystrophy photoreceptor degeneration.. Neuron.

[38] Swaroop A, Wang QL, Wu W, Cook J, Coats C (1999). Leber congenital amaurosis caused by a homozygous mutation (R90W) in the homeodomain of the retinal transcription factor CRX: direct evidence for the involvement of CRX in the development of photoreceptor function.. Hum Mol Genet.

[39] Furukawa T, Morrow EM, Li T, Davis FC, Cepko CL (1999). Retinopathy and attenuated circadian entrainment in Crx-deficient mice.. Nat Genet.

[40] Visel A, Thaller C, Eichele G (2004). GenePaint.org: an atlas of gene expression patterns in the mouse embryo.. Nucleic Acids Res.

[41] Yaylaoglu MB, Titmus A, Visel A, Alvarez-Bolado G, Thaller C (2005). Comprehensive expression atlas of fibroblast growth factors and their receptors generated by a novel robotic in situ hybridization platform.. Dev Dyn.

[42] Rhodes DR, Yu J, Shanker K, Deshpande N, Varambally R (2004). ONCOMINE: a cancer microarray database and integrated data-mining platform.. Neoplasia.

[43] Livingstone CD, Barton GJ (1993). Protein sequence alignments: a strategy for the hierarchical analysis of residue conservation.. Comput Appl Biosci.

[44] Huang J, Honda W (2006). CED: a conformational epitope database.. BMC Immunol.

[45] Bibb LC, Holt JK, Tarttelin EE, Hodges MD, Gregory-Evans K (2001). Temporal and spatial expression patterns of the CRX transcription factor and its downstream targets. Critical differences during human and mouse eye development.. Hum Mol Genet.

[46] Chen S, Wang QL, Nie Z, Sun H, Lennon G (1997). Crx, a novel Otx-like paired-homeodomain protein, binds to and transactivates photoreceptor cell-specific genes.. Neuron.

[47] Rath MF, Morin F, Shi Q, Klein DC, Moller M (2007). Ontogenetic expression of the Otx2 and Crx homeobox genes in the retina of the rat.. Exp Eye Res.

[48] Shyamsundar R, Kim YH, Higgins JP, Montgomery K, Jorden M (2005). A DNA microarray survey of gene expression in normal human tissues.. Genome Biol.

[49] Yanai I, Benjamin H, Shmoish M, Chalifa-Caspi V, Shklar M (2005). Genome-wide midrange transcription profiles reveal expression level relationships in human tissue specification.. Bioinformatics.

[50] Garraway LA, Sellers WR (2006). From integrated genomics to tumor lineage dependency.. Cancer Res.

[51] Ligon KL, Huillard E, Mehta S, Kesari S, Liu H (2007). Olig2-regulated lineage-restricted pathway controls replication competence in neural stem cells and malignant glioma.. Neuron.

[52] Gonzalez-Fernandez F, Lopes MB, Garcia-Fernandez JM, Foster RG, De Grip WJ (1992). Expression of developmentally defined retinal phenotypes in the histogenesis of retinoblastoma.. Am J Pathol.

[53] Czerwionka M, Korf HW, Hoffmann O, Busch H, Schachenmayr W (1989). Differentiation in medulloblastomas: correlation between the immunocytochemical demonstration of photoreceptor markers (S-antigen, rod-opsin) and the survival rate in 66 patients.. Acta Neuropathol.

[54] Jaffey PB, To GT, Xu HJ, Hu SX, Benedict WF (1995). Retinoblastoma-like phenotype expressed in medulloblastomas.. J Neuropathol Exp Neurol.

[55] Maraziotis T, Perentes E, Karamitopoulou E, Nakagawa Y, Gessaga EC (1992). Neuron-associated class III beta-tubulin isotype, retinal S-antigen, synaptophysin, and glial fibrillary acidic protein in human medulloblastomas: a clinicopathological analysis of 36 cases.. Acta Neuropathol.

[56] Kool M, Koster J, Bunt J, Hasselt NE, Lakeman A (2008). Integrated genomics identifies five medulloblastoma subtypes with distinct genetic profiles, pathway signatures and clinicopathological features.. PLoS ONE.

[57] Eberhart CG, Kratz J, Wang Y, Summers K, Stearns D (2004). Histopathological and molecular prognostic markers in medulloblastoma: c-myc, N-myc, TrkC, and anaplasia.. J Neuropathol Exp Neurol.

[58] Gulino A, Arcella A, Giangaspero F (2008). Pathological and molecular heterogeneity of medulloblastoma.. Curr Opin Oncol.

[59] Gilbertson RJ, Ellison DW (2008). The origins of medulloblastoma subtypes.. Annu Rev Pathol.

[60] Folpe AL (2002). MyoD1 and myogenin expression in human neoplasia: a review and update.. Adv Anat Pathol.

[61] Ligon KL, Alberta JA, Kho AT, Weiss J, Kwaan MR (2004). The oligodendroglial lineage marker OLIG2 is universally expressed in diffuse gliomas.. J Neuropathol Exp Neurol.

[62] Ponzoni M, Arrigoni G, Doglioni C (2007). New transcription factors in diagnostic hematopathology.. Adv Anat Pathol.

[63] Romeo S, Hogendoorn PC (2006). Brachyury and chordoma: the chondroid-chordoid dilemma resolved?. J Pathol.

[64] Glasker S, Li J, Xia JB, Okamoto H, Zeng W (2006). Hemangioblastomas share protein expression with embryonal hemangioblast progenitor cell.. Cancer Res.

[65] Li MK, Folpe AL (2004). CDX-2, a new marker for adenocarcinoma of gastrointestinal origin.. Adv Anat Pathol.

[66] de Jong J, Stoop H, Gillis AJ, van Gurp RJ, van de Geijn GJ (2008). Differential expression of SOX17 and SOX2 in germ cells and stem cells has biological and clinical implications.. J Pathol.

[67] Luo J, Solimini NL, Elledge SJ (2009). Principles of cancer therapy: oncogene and non-oncogene addiction.. Cell.

