# Supplementary figures and images for: CRX Is a Diagnostic Marker of Retinal and Pineal Lineage Tumors
Source: PLoS One. 2009 Nov 20;4(11):e7932. doi: 10.1371/journal.pone.0007932 (PMC2775954; doi:10.1371/journal.pone.0007932)

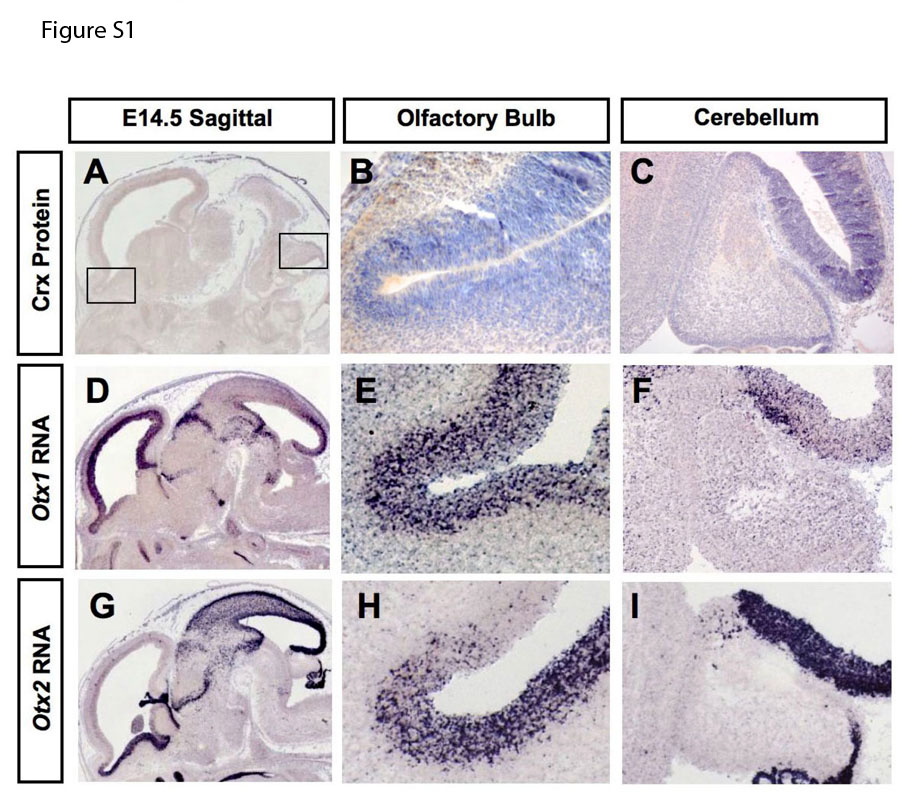

Supplement: Figure S1 — Comparison of H120 antibody staining (Crx) with RNA expression pattern of Otx1 and Otx2. Immunohistochemistry for Crx using H120 antibody in E14.5 mouse (A, B, C, brown DAB) shows no overlap with the RNA expression pattern (purple, BMPurple) of Otx1 (D, E, F) or Otx2 (G, H, I), including areas with high level Otx expression such as the olfactory bulb and cerebellum. This suggests that H120 is specific for Crx and does not recognize the closest predicted family members. (2.18 MB TIF) [file pone.0007932.s001.tif]

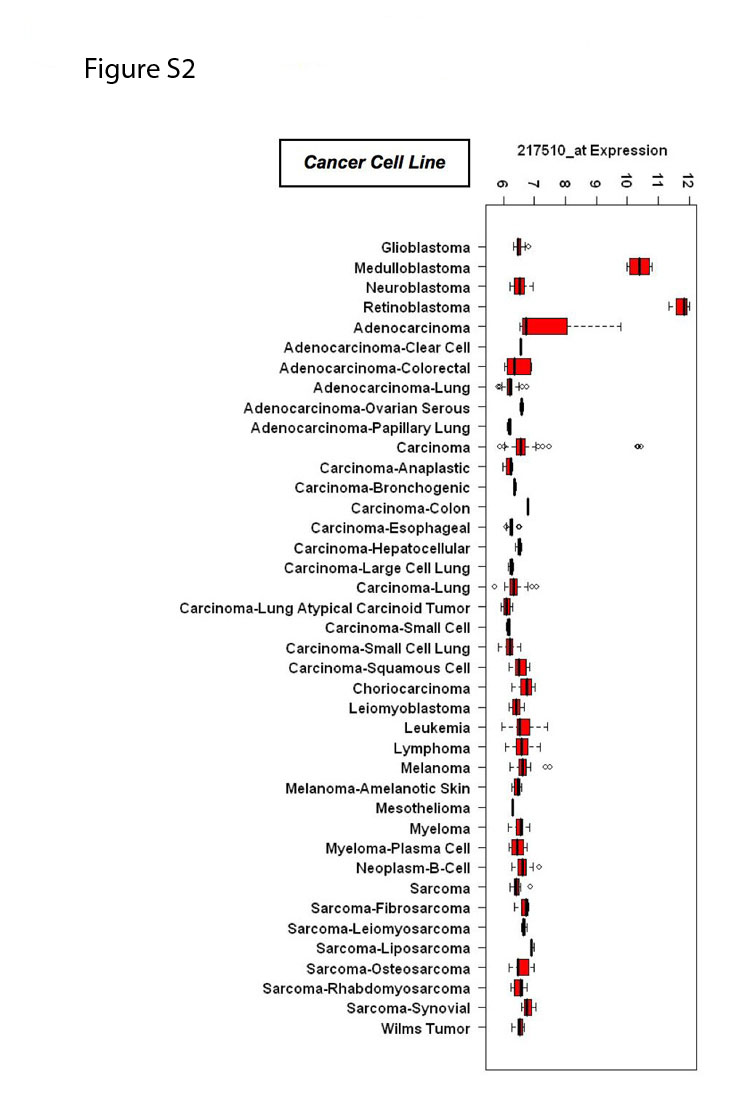

Supplement: Figure S2 — Meta-analysis of expression profiling in cancer cell lines shows high level expression of CRX in retinoblastoma and medulloblastoma cell lines. The Glaxo-Smith-Kline human cancer cell line dataset was normalized and analyzed for expression of the CRX specific probeset 217510. Highest expression was seen in two medulloblastoma cell lines and the single retinoblastoma cell line in the dataset. Most other cell lines showed little to no expression of CRX. (2.52 MB TIF) [file pone.0007932.s002.tif]
